# Procedural efficiency is enhanced combining the pentaspline pulsed field ablation catheter with three-dimensional electroanatomical mapping system for pulmonary vein isolation

**DOI:** 10.1007/s10840-024-01846-3

**Published:** 2024-06-10

**Authors:** Alessio Falasca Zamponi, Jens Olson, Sergej Scheel, Anders Englund, Raffaele Scorza, Fariborz Tabrizi

**Affiliations:** 1 Capio Arytmicenter Stockholm AB, Stockholm, Sweden; 2 Department of Clinical Science and Education, Division of Cardiology, Karolinska Institutet, South Hospital, Stockholm, Sweden

**Keywords:** Atrial fibrillation, Pulmonary vein isolation, Pulsed field ablation, 3D-EAM

## Abstract

**Background:**

Pulsed field ablation (PFA) offers a safe, non-thermal alternative for pulmonary vein isolation (PVI) in patients with atrial fibrillation (AF). Currently, the pentaspline PFA-system relies heavily on fluoroscopy for catheter manipulation, which poses challenges due to the complexity of left atrium anatomy. Incorporating three-dimensional electroanatomical mapping (3D-EAM) could improve procedural efficiency reducing dependency on fluoroscopy guidance. This study aims to evaluate the effects of integration of 3D-EAM with PFA during PVI.

**Methods:**

Between September 2022 and December 2023, we retrospectively enrolled 248 patients with paroxysmal or persistent AF undergoing PVI at our center using the pentaspline PFA catheter. The control group (*n* = 104) received conventional PFA with fluoroscopic guidance alone, while the intervention group (*n* = 144) underwent PVI with PFA with 3D-EAM integration. Primary outcomes were procedural time, fluoroscopy time (FT), and dose area product (DAP). Secondary endpoints included acute procedural success and incidence of periprocedural complications.

**Results:**

In the 3D-EAM-PFA group, procedural time was 63.3 ± 14.3 min, compared to 65.6 ± 14.9 min in the control group (*p* = 0.22). The 3D-EAM group experienced significantly reduced FT (9.7 ± 4.4 min vs. 16.7 ± 5.2 min) and DAP (119.2 ± 121.7 cGycm^2^ vs. 338.7 ± 229.9 cGycm^2^) compared to the control group, respectively (*p* < 0.001). Acute procedural success was achieved in all cases. No major complications were observed in either group.

**Conclusion:**

Integration of 3D-EAM with the pentaspline PFA catheter for PVI in AF treatment offers a promising approach, with significantly reduced fluoroscopy exposure without compromising procedural time and efficacy.

**Graphical abstract:**

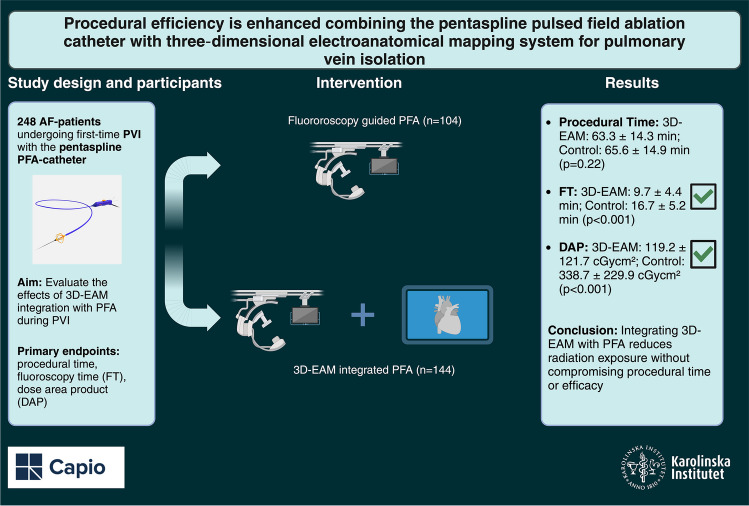

**Supplementary Information:**

The online version contains supplementary material available at 10.1007/s10840-024-01846-3.

## Introduction

Atrial fibrillation (AF) is the most common sustained cardiac arrhythmia, causing a significant global health burden with a prevalence ranging from 2 to 4% among adults [1]. This arrhythmia is associated with significantly increased risk of stroke, heart failure, and premature death [2–4]. Over the years, pulmonary vein isolation (PVI), aimed at electrically isolating the pulmonary veins (PVs) from the left atrium (LA) to prevent AF symptoms, has emerged as a first-line treatment. Until recently, PVI has been achieved using thermal energy sources like radiofrequency or cryoablation. However, these modalities come with their own set of challenges, including potential for serious complications such as PV stenosis (≤ 1%), phrenic nerve palsy (up to 4%), and atrio-esophageal fistula (< 0.1–0.25%) [5–7].

Pulsed field ablation (PFA) is a promising and novel non-thermal tissue-specific ablation modality that utilizes ultrarapid electric pulses to create transmural tissue lesions preferentially in myocardial tissue [8, 9]. Recent studies have demonstrated that PVI could be achieved using PFA without any major adverse effects associated with thermal ablation. The safety and efficiency of PFA in AF ablation have been reported in previous studies [10–13].

The visualization and assessment of the pentaspline PFA catheter in the LA, including the ostia of PVs, is routinely carried out under fluoroscopy guidance. This method can occasionally be challenging due to factors such as the complex anatomy of the PVs, especially regarding the right-sided ones [14], and the importance of achieving effective tissue contact [15]. In this context, integrating three-dimensional electroanatomical mapping (3D-EAM) with PFA could potentially overcome these challenges.

With this study, we aim to compare 3D-EAM integrated PFA to conventional PFA with fluoroscopic guidance alone during PVI procedures.

## Methods

We retrospectively assessed the outcome of 144 patients diagnosed with paroxysmal or persistent AF who underwent PVI at our center. All patients were treated with PVI for the first time, and the procedure was performed with the pentaspline PFA catheter integrated with 3D-EAM. Patients underwent the combined PFA/3D-EAM procedure between April and December 2023. For patients 1 to 41, a two-step mapping procedure of the LA with the coronary sinus (CS) catheter and the pentaspline PFA catheter was performed. For patients 42 to 144, the CS catheter was omitted, and mapping was exclusively conducted using the PFA catheter. A control group of 104 individuals who had previously received conventional PFA without the use of 3D-EAM was also included in the analysis. The measured outcomes were procedural time, fluoroscopy time (FT) and dose, expressed as dose area product (DAP). Procedural time was defined as time from femoral vein puncture to removal of the catheters sheaths out of the femoral vein. Additionally, acute procedural success and the occurrence of periprocedural complications were analyzed. Acute procedural success was defined as entrance- and exit-block for all PVs.

Informed patient consent was obtained. The study was approved by the Swedish National Ethics Committee (Dnr 2023–08026-01) and complied with the Declaration of Helsinki.

### Ablation procedure

All procedures were performed by three senior electrophysiologist consultants at our center. Each of these electrophysiologists has an extensive background within invasive electrophysiology (> 15 years), and they have been performing PVI with PFA since August 2022.

The patients underwent the procedures under uninterrupted oral anticoagulant therapy. Transesophageal echocardiography (TEE) was performed for the exclusion of intracardiac thrombus in all patients with a CHADS-VASc score of ≥ 1 point or presenting with AF at the time of the procedure. Antiarrhythmic drugs were discontinued five half-lives in advance, with the exception of amiodarone, which was stopped 30 days before ablation. Each procedure was carried out following a predetermined deep sedation plan overseen by a nurse anesthetist (Supplementary material 1). An anesthesiologist was available on call to address any sedation-related issues.

Venous access was established through two femoral vein punctures, and a decapolar CS catheter was advanced in the CS. A transseptal puncture was performed using an SL1 sheath (Abbott Laboratories, Chicago, IL, USA) and a transseptal needle (TSX, Boston Scientific, Natick, MA, USA). Via the SL1 sheath, the decapolar catheter was advanced into the LA to create a three-dimensional electroanatomic map using the EnsiteX system (Abbott Laboratories, Chicago, IL, USA). Subsequently, the decapolar catheter was repositioned in the CS, preferably in a ventricular branch for enabling ventricular pacing.

The PFA system consists of three components: a proprietary generator (FARASTAR, FARAPULSE, Boston Scientific, Natick, MA, USA) providing a multi-channel high-voltage pulsed field waveform, an ablation catheter (FARAWAVE) available in diameters of 31 or 35 mm, and a 13-F steerable sheath (FARADRIVE). The FARAWAVE catheter is equipped with five splines, with four electrodes per spline, capable of deploying in a flower or basket configuration.

The SL1 sheath was replaced with the FARADRIVE sheath, and the FARAWAVE catheter was introduced into the LA. After transseptal puncture, a dose of Heparin (100 IU/kg) was administered with the aim of achieving an activated clotting time (ACT) greater than 330 s. The intracardiac electrograms (EGMs) and surface electrograms were recorded at a speed of 100 mm/s (Sensis, Siemens, Erlangen, Germany).

The catheter was maneuvered over a guidewire until the splines made circumferential contact with the PV ostia/antra. The position and contact between the catheter and PV ostium/antrum were verified using fluoroscopy and the EnsiteX system. Visual assessment of the catheter was performed in basket and flower positions through EAM, with fluoroscopy serving as a guide when further catheter advancement was not feasible. No contrast was used.

Ablative energy was administered from all electrodes, with the third electrode on each spline also capable of recording EGMs. Each spline was color-coded in the EnsiteX system, corresponding to the same EGM-color on the EP-recording system, enabling correlation of the EGM with spatial information related to the placement of each spline. The catheter was rotated after every two applications to ensure circumferential coverage of the PV ostial and antral regions. Four applications were delivered with the catheter in the basket configuration and an additional four in the flower configuration, each application consisting of five pulses.

Patients with left common ostium (LCO) received therapy at the level of the common antrum with two sets of application delivered, one with the guide wire in a superior branch and one with the guide wire in an inferior one. Additional applications were delivered at the discretion of the operator.

To prevent the occurrence of bradycardia or asystole during ablation, atropine at a dose of 0.5 mg was administered prophylactically prior to ablation. In instances where a vagal reaction still induced bradycardia or asystole during ablation, ventricular pacing was implemented as a management strategy.

Pre- and post-ablation EGMs were recorded using the FARAWAVE catheter, positioned in the PV. Isolation after ablation was confirmed by the absence of PV potential post-ablation. Pacing at bipolar pairs was carried out using the ablation catheter in the PV to confirm the exit block.

### Visualization of FARAPULSE and guidewire on the EAM system (patients 1–41)

**Fig. 1:**
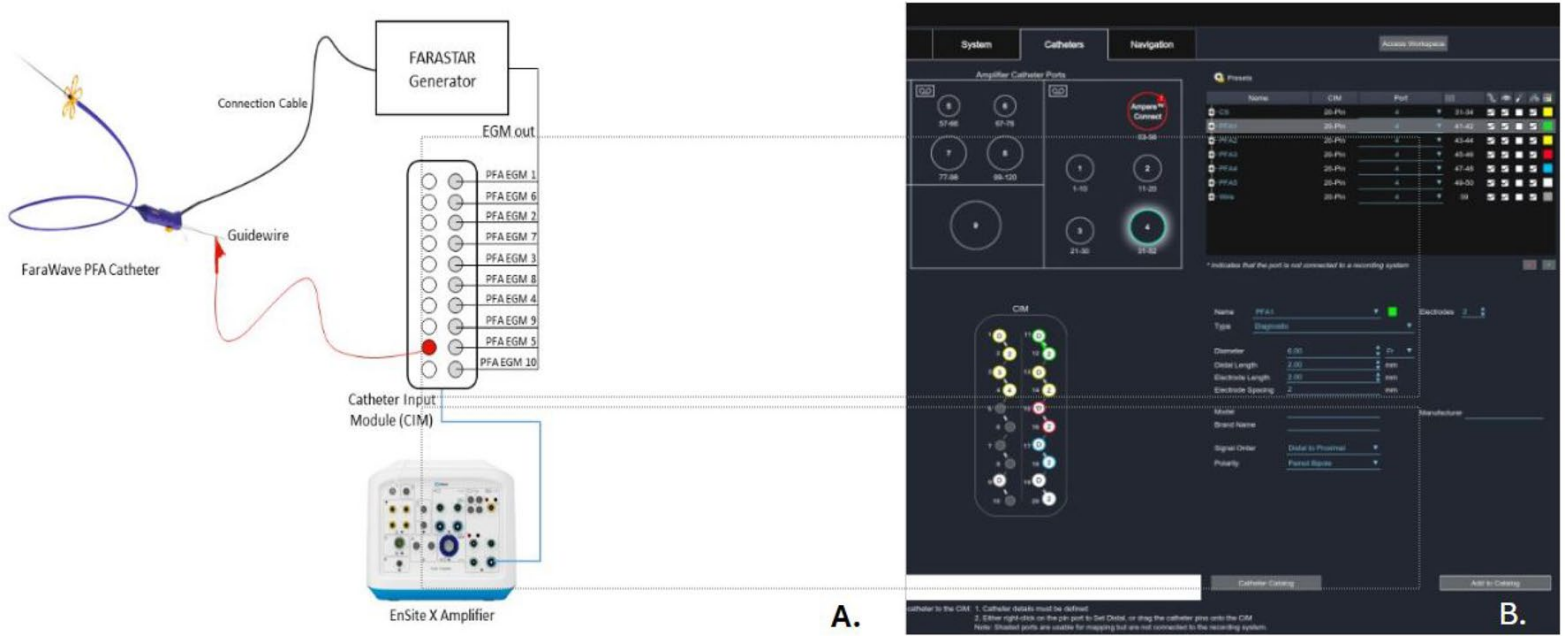
**A** Schematic connection diagram of the Farawave Catheter and guidewire to the EnsiteX Mapping System. **B** Chatheter/guidewire setup in the EnsiteX system

**Fig. 2:**
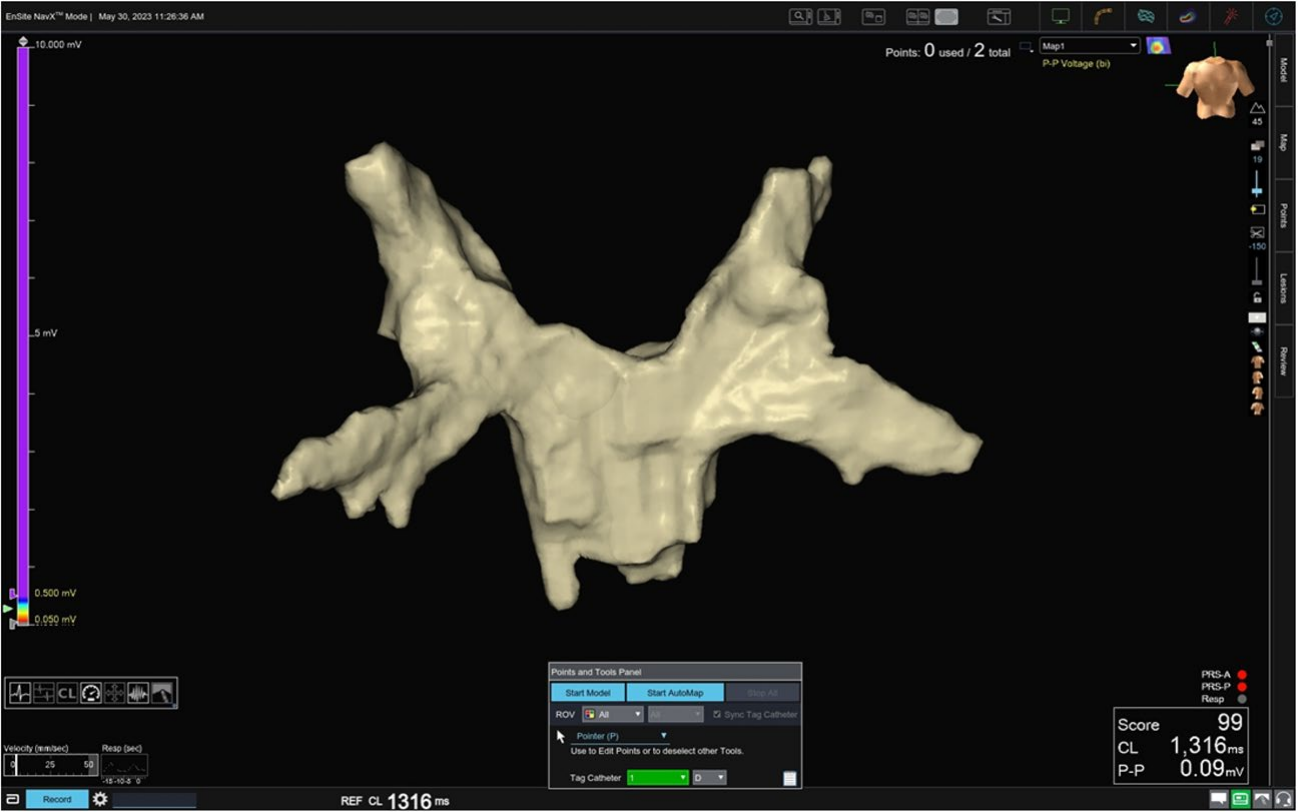
Initial “raw” electroanatomic map (postero-anterior, PA view) of the LA with emphasis on PV locations sampled using the distal four electrodes of the decapolar CS catheter

The CS-catheter, the FARAWAVE catheter and the guidewire were subsequently linked to the catheter interface module (CIM) on the EnsiteX system, as depicted in Fig. 1. Using the NavX modality of the EnsiteX system, a two-stage mapping of the LA was conducted. After transseptal access to the LA, a preliminary three-dimensional map was created using the decapolar CS-catheter, providing a basic anatomical outline with emphasis on the location of the PVs (Fig. 2). This procedure was rather quick, usually taking 3–5 min to complete. For this phase, we used only the four distal electrodes of the CS-catheter to prevent over-sampling from other structures such as the right atrium. This preliminary mapping, termed “raw mapping” within our team served as the basis upon which a more detailed and precise mapping was created with the PFA catheter.

**Fig. 3:**
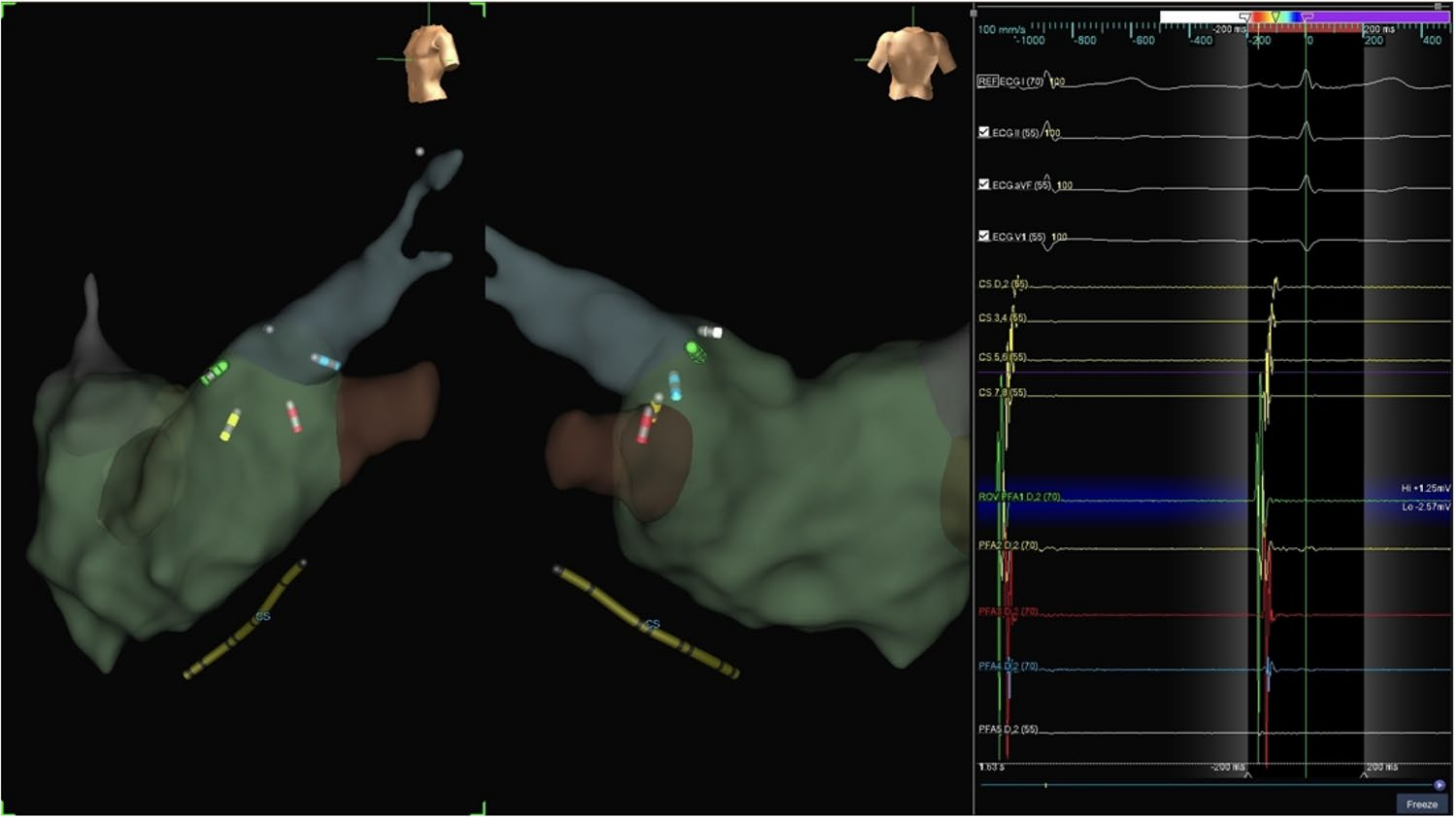
Color-coded representation of the pentaspline PFA catheter deployed at the left superior pulmonary vein (LSPV) during the PVI procedure in the EnsiteX system, LAO respective PA view. The PFA catheter is represented as five distinct bipoles each representing a spline. The color of each spline corresponds to the same EGM-color on the EP-recording system. The tip of the guidewire is represented as an orb

**Fig. 4:**
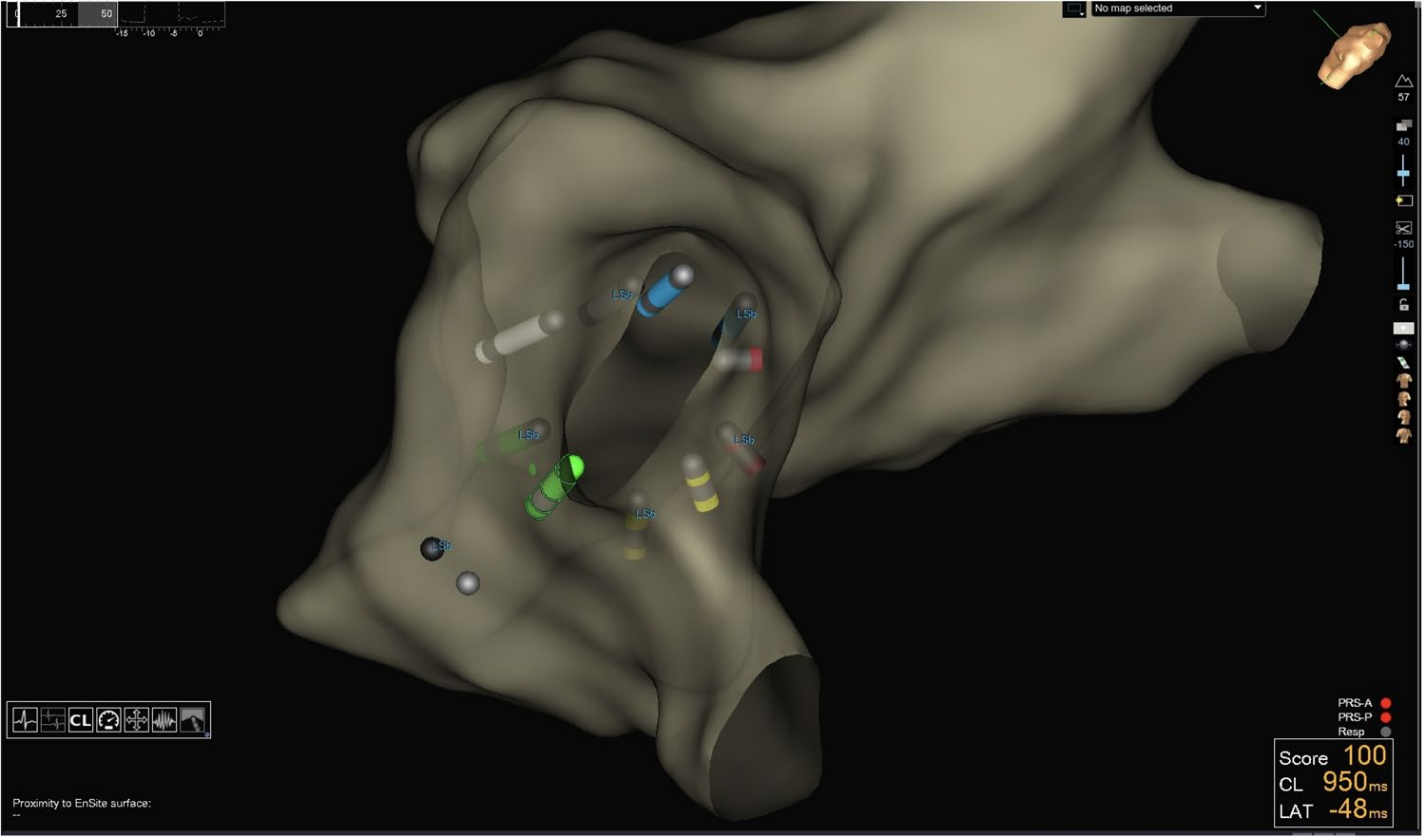
Illustration of the “shadow” visualization technique in the EnsiteX system, showcasing the positioning of the pentaspline PFA catheter after rotation for ablation coverage around the PV ostium. The PFA catheter is deployed at the LSPV in basket configuration, and left lateral superior view is used for optimal catheter visualization during rotation

When subsequently the PFA catheter was deployed within the LA, its splines were represented on the EAM as five distinct bipolar catheters (Fig. 3). Within each bipole, the distal electrode was represented by the interpolation of electrode one, two, and four of each spline, while the distal electrode was represented by the subequatorial electrode (third electrode) of each spline. This phase of our procedure involved continuous anatomy sampling from the five bipoles, which contributed to refine the map of the LA. During ablation, prior to each rotation, we captured a shadow representation of each bipole/spline. The shadow image served as a visual guide, enabling us to align the catheter’s subsequent placement, ensuring that each section of the PV ostium and antrum received uniform exposure to the ablation energy (Fig. 4).

For guidewire visualization, the guidewire was connected to the recording system (pin 9 on the CIM, Fig. 1) using pacing cables equipped with alligator clips (5833, Medtronic, Minneapolis, MN, USA). This connection effectively transformed the guidewire into an additional unipolar electrode. As a result, the tip of the guidewire was visualized as an orb on the EAM system (Fig. 3). This approach for guidewire visualization builds upon the experience described by Cronin et al. (2013) [16] in the setting of cryoballoon ablation of atrial fibrillation. The position of the guidewire was in the initial phase of the study cross-referenced by the operator with its location as indicated by orthogonal fluoroscopy in the right anterior oblique (RAO) and left anterior oblique (LAO) views.

### Visualization of FARAPULSE and guidewire on the EAM system (patients 42–100)

For patients 42 to 100, anatomy sampling with the CS catheter was intentionally omitted. This decision was made based on the experience and confidence gained with the previous patients and with the aim of reducing potential risks associated with the introduction and manipulation of an additional catheter in the LA. Mapping was exclusively performed using the PFA catheter as described above. The ablation procedure remained otherwise identical.

### Control group

The control group consisted of individuals with AF who had previously (September 2022–April 2023) undergone conventional PFA for PVI without the integration of 3D-EAM guidance. To minimize biases related to the initial learning phase, we included cases starting from late September 2022, excluding those performed immediately after the introduction of the method at our clinic in August 2022. A total of seventeen patients were excluded due to this consideration. All AF ablation procedures in the two groups were performed by the same three operators.

### Statistical analysis

All analyses were conducted in R, version 4.3.2 (R Foundation for Statistical Computing, Vienna, Austria). Distributions of continuous variables were tested for normality using the Kolmogorov–Smirnov test. Continuous variables are expressed as mean ± standard deviation, while categorical variables are presented as counts and percentages. To compare continuous variables with a normal distribution, we utilized the *t*-test, whereas for categorical variables, we employed either χ 2 or Fisher’s exact test as appropriate. Linear regression analysis was performed to assess trends over time. A *p* value < 0.05 was considered threshold for statistical significance.

## Results

Patients’ characteristics are presented in Table 1, while procedure-related parameters are summarized in Table 2. In both groups, there was a majority of male patients with paroxysmal AF and a low burden of comorbidities.

**Table 1:** Patients’ characteristics

	All (*n* = 248)	Control group (*n* = 104)	3D-EAM-PFA group (*n* = 144)	*p* value
Age, years	60.6 ± 10.7	60.6 ± 10.2	60.6 ± 11.1	0.97
Sex (male), *n* (%)	191 (77.0)	79 (76.0)	112 (77.8)	0.53
BMI	26.6 ± 3.2	26.8 ± 3.4	26.4 ± 3.1	0.31
Hypertension, *n* (%)	78 (31.5)	28 (26.9)	50 (34.7)	0.53
Diabetes mellitus, *n* (%)	12 (4.8)	4 (3.9)	8 (5.6)	0.75
Stroke/TIA, *n* (%)	6 (2.4)	2 (1.9)	4 (2.8)	0.67
Vascular disease, *n* (%)	6 (2.4)	3 (2.9)	3 (2.1)	0.69
AF Paroxysmal/persistent, %	134/114 (54.0/46.0)	60/44 (57.7/42.3)	74/70 (51.4/48.6)	0.33
LA volume, *n* (%)				
Normal range (16–34 ml/m^2^)	142 (57.3)	66 (63.5)	76 (52.8)	0.25
Mildly abnormal (35–41 ml/m^2^)	77 (31.1)	27 (26.0)	50 (34.7)	0.18
Moderately abnormal (42–48 ml/m^2^)	27 (10.9)	11 (10.6)	16 (11.1)	1.00
Severely abnormal (> 48 ml/m^2^)	2 (0.8)	0.0 (0)	2 (1.4)	0.63
CHA2DS2-VASc score, *n* (%)				
0	87 (35.1)	43 (41.4)	44 (30.6)	0.08
1	68 (27.4)	23 (22.1)	45 (31.3)	0.12
2	49 (19.8)	21 (20.2)	28 (19.4)	1.00
3	28 (11.3)	9 (8.7)	19 (13.2)	0.31
4	10 (4.0)	5 (4.8)	5 (3.5)	0.75
≥ 5	6 (2.4)	3 (2.9)	3 (2.1)	0.70
Cardiomyopathy, *n* (%)				
Ischemic	10 (4.0)	5 (4.8)	5 (3.5)	0.60
Non-ischemic	7 (2.8)	4 (3.9)	3 (2.1)	0.41
Tachycardiomyopathy	8 (3.2)	2 (1.9)	6 (4.2)	0.32
LVEF^a^, *n* (%)				
Normal range (≥ 50%)	223 (89.9)	97 (93.3)	126 (87.5)	0.13
Mildly reduced (41–49%)	21 (8.5)	6 (5.8)	15 (10.4)	0.19
Moderately reduced (30–40%)	3 (1.2)	1 (1.0)	2 (1.4)	0.76
Severely reduced (< 30%)	1 (0.4)	0.0 (0)	1 (0.7)	0.39
Class I or III AAD, *n* (%)	153 (61.7)	58 (55.8)	95 (66.0)	0.10
CIED, *n* (%)	5 (2.0)	1 (1.0)	4 (2.8)	0.31

For quantitative variables, values are expressed as mean ± SD. Categorical variables are presented as counts and percentages. *BMI*, body mass index; *TIA*, transient ischemic attack; *AF*, atrial fibrillation; *LA*, left atrial; *CHA2DS2-VASc*, congestive heart failure, hypertension, age ≥ 75 (doubled), diabetes, stroke (doubled), vascular disease, age 65 to 74 and sex category (female); *LVEF*, left ventricular ejection fraction ^a^ cut-offs defined according to the Swedish Catheter Ablation Registry; *AAD*, antiarrhythmic drugs; *CIED*, cardiac implantable electrical devices

**Table 2:** Procedural parameters

	All (n = 248)	Control group (n = 104)	3D-EAM-PFA group (n = 144)	*p* value
Procedural time, min	64.2 ± 14.6	65.6 ± 14.9	63.3 ± 14.3	0.22
FT, min	12.6 ± 5.9	16.7 ± 5.2	9.7 ± 4.4	** < 0.001**
DAP, cGy cm^2^	211.3 ± 205.9	338.7 ± 229.9	119.2 ± 121.7	** < 0.001**
PFA applications, *n*	34.7 ± 4.0	34.4 ± 4.1	34.8 ± 3.8	0.44
RF ablation touch-up, *n* (%)	0 (0)	0 (0)	0 (0)	1.00
Same day discharge, *n* (%)	168 (67.7)	67 (64.4)	101 (70.1)	0.34
PV anatomy, *n* (%)				
Four separate veins	219 (88.3)	96 (92.3)	123 (85.4)	0.10
LCO	23 (9.3)	7 (6.7)	16 (11.1)	0.24
Right intermediate	6 (2.4)	1 (1.0)	5 (3.5)	0.20

Statistically significant comparisons are indicated in bold. *FT*, fluoroscopy time; *DAP*, dose area product; *RF*, radiofrequency; *PV*, pulmonary vein; *LCO*, left common ostium

The procedural time was 63.3 ± 14.3 min in the 3D-EAM group and 65.6 ± 14.9 min in the control group. This difference did not reach statistical significance (*p* = 0.22).

In the 3D-EAM-PFA cohort, the mean FT and mean DAP showed significant reductions compared to the control group. More specifically, the mean FT was 9.7 ± 4.4 min in the 3D-EAM group compared to 16.7 ± 5.2 min in the controls. Mean DAP was 119.2 ± 121.7 cGy cm^2^ compared to 338.7 ± 229.9 cGy cm^2^. Observed reductions were statistically significant (*p* < 0.001) for both FT and DAP.

**Fig. 5:**
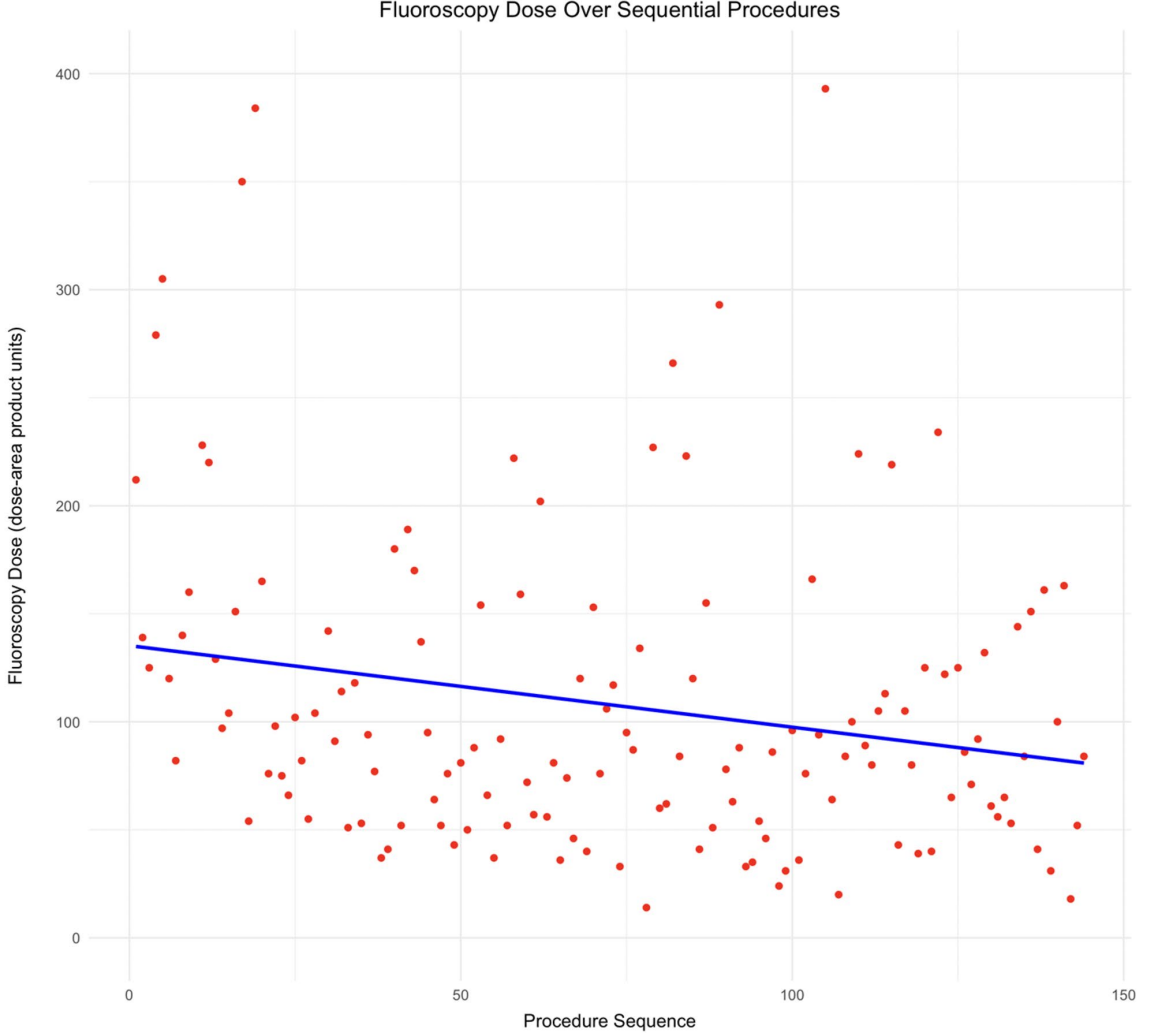
Decreasing trend in DAP over the sequence of procedures within the 3D-EAM-PFA cohort. Linear regression analysis was conducted to assess the trend, with two outliers excluded to promote better visualization

Further analysis of DAP within the 3D-EAM-PFA revealed a statistically significant decreasing trend in DAP over the sequence of procedures (Fig. 5). Similar analysis on procedural time and fluoroscopy time did not show any statistically significant decreasing trend.

### Safety and efficacy

During this study, 975 PVs were targeted in 248 patients. All of the PVs were successfully isolated with PFA only. Additional PFA applications after the initial set of 32 applications were administered in 44 out of 104 patients in the control group (42.3%) and 78 out of 144 patients in the intervention group (54.2%). The difference in the proportion of patients receiving additional PFA applications between the control group and the intervention group was not statistically significant (*p* = 0.086). No touch-up thermal ablations were needed.

In the control group, there were two instances of minor bleeding at the puncture site, requiring pressure bandage, without the need for other intervention or extended hospital stay. One major bleeding was registered in the control group, necessitating surgical intervention at the puncture site. In the intervention group, one patient experienced post-procedural fever, which resolved spontaneously within 24 h. Blood cultures conducted in response to the fever yielded negative results. No instances of pericardial effusion or tamponade, ST-segment elevation, phrenic nerve palsy, air embolism, or death were observed during the study.

## Discussion

Our study investigates the safety and efficacy of a novel technique that integrates the pentaspline PFA catheter with the EnsiteX mapping system for PVI procedure.

For patients 1–41 in the 3D-EAM-PFA-group, we created a foundational map of the LA and PVs using the decapolar CS catheter. This initial map could be created safely and quickly with minimal need for fluoroscopy. Moreover, it served as a valuable tool in building confidence with our method. As our study progressed, however, we observed that relying solely on the PFA catheter for mapping streamlined the workflow. Additionally, this approach eliminated the risks associated with inserting additional hardware into the LA.

By strategically combining 3D-EAM with the information from fluoroscopy, we believe that our technique allows a better positioning and rotation of the PFA catheter. Guiding the placement of the catheter according to the unique anatomy of each patient, it potentially enhances the precision of the ablation, promoting a circumferential treatment around the PVs. For clarity and intuitiveness, we color-coded the splines to match the recorded EGMs, providing a clearer representation of the position of the PFA catheter within the LA (Fig. 3).

In our workflow, before each rotation of the catheter, we utilized the system to capture a “shadow” representation of its position. This “shadow” visualization played a crucial role in ensuring that each subsequent rotation of the PFA catheter was optimally aligned, thus helping in obtaining a more uniform ablation around the vein (Fig. 4). We believe that this approach represents a significant improvement over conventional technique, which often relies on estimations and can result in inconsistent ablation delivery due to the complex and variable anatomy of the PVs.

Recent advancements in the EnsiteX system have introduced a new software module that provides visual representation of the FARAWAVE catheter in both basket and flower configurations. Although this enhancement was not available at the time of our study, its introduction holds promise to further improve catheter navigation and procedural efficacy.

The integration with 3D-EAM offers additional advantages. A standout feature is real-time visualization of the guidewire, ensuring that both the guidewire and the catheter are navigated along the planned path. This reduces the risk of inadvertent contact with sensitive structures such as the left trial appendage (LAA). The ability to track the guidewire holds therefore the potential to reduce reliance on fluoroscopy and possibly decrease the likelihood of procedural complications.

In our study, we did not perform any voltage mapping of the left atrial substrate nor any additional ablation procedures such as posterior wall isolation. Nevertheless, the integration of 3D-EAM with PFA opens interesting avenues for future research, particularly in terms of atrial substrate modification. The ability to accurately identify and ablate pathological atrial substrates could significantly improve treatment outcomes for patients with AF, especially those with persistent forms. However, as the current pentaspline PFA catheter lacks validation for such applications, future studies should consider bridging this gap.

In our comparative analysis, we observed a substantial reduction in fluoroscopy usage between the control group and the 3D-EAM group, indicating decreased dependence on fluoroscopy as operators gained proficiency with the system. This proficiency contributed to a 41.9% decrease in FT and a 64.8% decrease in DAP when comparing the two groups. Further analysis of DAP within the 3D-EAM-PFA group revealed a statistically significant decreasing trend over the sequence of procedures (Fig. 5). This trend suggests a learning curve in radiation exposure management within the cohort. This not only benefits patients by reducing radiation exposure but also creates a safer work environment for catheterization laboratory staff due to reduced occupational radiation exposure.

Furthermore, in our intervention cohort, 3D-EAM replaced the need for computed tomography (CT) scans or PV angiography traditionally used to discern the anatomical layout of the LA and the PVs.

This approach offers several advantages, including a reduction in ionizing radiation exposure, elimination of the need for contrast media, and potential acceleration of the timeline from diagnosis to intervention, which is particularly valuable in healthcare systems facing resource limitations or significant patient backlogs.

Our study has several limitations, being a single-center study with a retrospective design. However, the choice of design took into consideration ethical issues in prospectively randomizing patients to a method that could potentially expose them to excessive radiation. To mitigate potential biases from the initial learning phase in the control group, our analysis commenced in late September 2022. This precaution was necessary as the method was introduced at our clinic in August 2022, and including early cases might have skewed the results due to the associated learning curve.

It is important to emphasize that although our results are promising, they are based on preliminary data obtained from a single-center experience. Wider validation from multicenter studies is essential to confirm and extend our results. To further explore the potential benefits hypothesized in our study, a follow-up study focusing on the clinical outcomes of patients treated with this integrated technique would be beneficial. This subsequent research should aim to evaluate the long-term efficacy of the lesions created by the novel approach, particularly in terms of patient symptom relief, quality of life improvements, and AF recurrence rates. Moreover, conducting cost-effectiveness analyses could assist in determining whether the potential advantages of this technique justify its expenses, a critical factor to weigh when considering its widespread clinical implementation. It is important to note, however, that the cost increase for this technique is rather marginal, as it utilizes the NavX, the impedance-based modality of EnsiteX system, and avoids the use of expensive proprietary magnetic-enabled catheters.

In our patient cohort, intracardiac echocardiography (ICE) was not utilized in any of the instances. Recent research [17] has shown that while ICE may not offer significant advantages in procedural parameters, it remains an intriguing tool for enhancing safety, visualizing anatomical structures of the LA, and ensuring tissue contact during ablation procedures. Integrating ICE with 3D-EAM and PFA could potentially offer synergistic benefits, particularly in complex cases where precise visualization and catheter manipulation are crucial.

Looking ahead, it is clear that second-generation PFA catheters will prominently rely on 3D-EAM as the primary modality for visualizing these devices within the heart. In this context, our study offers a glimpse into this future.

## Conclusion

This study provides evidence of the feasibility of the integration of 3D-EAM with the pentaspline PFA catheter for PVI procedures in patients with AF. While our findings indicate that procedural time may not significantly benefit from 3D-EAM guidance, it is noteworthy that both FT and DAP are substantially reduced.

## Supplementary Information

Below is the link to the electronic supplementary material.

Supplementary file1 (DOCX 11 KB)

## Data Availability

The data that support the findings of this study are available upon reasonable request.
