# Supplementary material for: Procedural efficiency is enhanced combining the pentaspline pulsed field ablation catheter with three-dimensional electroanatomical mapping system for pulmonary vein isolation
Source: J Interv Card Electrophysiol. 2024 Jun 10;67(9):1993–2001. doi: 10.1007/s10840-024-01846-3 (PMC11711628; doi:10.1007/s10840-024-01846-3)
Supplement: Supplementary file 1 — Supplementary file1 (DOCX 11 KB) [file 10840_2024_1846_MOESM1_ESM.docx]

**Sedation protocol**

1. Pre-electrophysiology lab

- No sedatives administered at this stage

1. Femoral puncture to trans-septal puncture

- Fentanyl 0.5 – 1 μg/kg iv

1. PVI

- Propofol 3-6 mg/kg/hour
- Oxygen 15 L/min administered through face mask
- Guedel tube with spontaneous respiration
